# Evaluating the Performance of Video-Based Automated Passenger Counting Systems in Real-World Conditions: A Comparative Study

**DOI:** 10.3390/s23187719

**Published:** 2023-09-07

**Authors:** Cristina Pronello, Ximena Rocio Garzón Ruiz

**Affiliations:** Interuniversity Department of Regional and Urban Studies and Planning, Politecnico di Torino, 10125 Turin, Italy; ximena.garzon@polito.it

**Keywords:** public transport, automated passenger counting (APC), accuracy, performance, camera-based systems, optical systems, low-cost APC system, intelligent transport systems

## Abstract

Automatic passenger counting (APC) systems in public transport are useful in collecting information that can help improve the efficiency of transport networks. Focusing on video-based passenger counting, the aim of this study was to evaluate and compare an existing APC system, claimed by its manufacturer to be highly accurate (98%), with a newly developed low-cost APC system operating under the same real-world conditions. For this comparison, a low-cost APC system using a Raspberry Pi with a camera and a YOLOv5 object detection algorithm was developed, and an in-field experiment was performed in collaboration with the public transport companies operating in the cities of Turin and Asti in Italy. The experiment shows that the low-cost system was able to achieve an accuracy of 72.27% and 74.59%, respectively, for boarding and alighting, while the tested commercial APC system had an accuracy, respectively, of 53.11% and 55.29%. These findings suggest that current APC systems might not meet expectations under real-world conditions, while low-cost systems could potentially perform at the same level of accuracy or even better than very expensive commercial systems.

## 1. Introduction

Intelligent Transport Systems (ITS) contribute to a safer, more efficient, and environmentally friendly transport network. They make different aspects of urban mobility smarter, especially strategic planning, where they have made automated transport data possible. They can improve the operations of the entire transport network, leading to a better coordination of physical flows and resources [1,2].

The role of public transport is important in the context of smart mobility since it can reduce car traffic flows. Transport companies seek to increase efficiency by collecting information from a variety of sources [3]. A number of companies have chosen to install automatic passenger counting (APC) systems in their vehicles in order to better estimate vehicle occupancy and to have more accurate information regarding the boarding and alighting of users. Different commercial systems using different technologies are available: weight-based systems, mobile device-based systems, video imaging, and other optical and infrared technologies. In recent years, with the rapid development of new technologies such as artificial intelligence (AI) and deep learning (DL) algorithms, there has been a greater focus on camera-based systems; this is the case not only in transport, but also in the retail, manufacturing and health sectors [4,5,6,7].

APC optical solutions include video counting systems and stereoscopic camera implementations. These technologies measure passenger volumes and the boarding and alighting of passengers. In most cases, a number of different algorithms are used for detecting motion, estimating the direction of the identified object, and confirming the existence of a moving passenger [8]. These APC systems are usually mounted above the doors to count passengers entering and leaving the vehicle; however, alternative solutions for counting passengers have been developed by employing Closed-Circuit Television (CCTV) cameras placed within the vehicles. The prevalent characteristic in both approaches is the zenithal positioning of the cameras, i.e., above passengers’ heads [9]. In Europe, seven major companies produce and sell APC systems, either as manufacturers or system integrators. The main products available on the market are shown in Table 1. Manufacturers produce and sell hardware components for the APC systems, while system integrators buy APC devices and incorporate them into commercial packages, offering services such as video surveillance and smart ticketing in addition to the devices themselves.

Some of these companies meet the VDV 457-2 accuracy requirements [10,11]. VDV stands for Verband Deutscher Verkehrsunternehmen, the association of German transport companies that publishes recommendations and general advice concerning the implementation of APC systems in different vehicles so as to ensure the quality and uniformity of the data collected and consequently the reliability of projections based on these data. VDV 457-2 sets out rules for verifying and certifying counting accuracy [12]. Nonetheless, no comprehensive universal standard for measuring passengers onboard in public transport has so far been established. As an illustration of this absence of a common standard, countries including the United Kingdom, the United States, and Australia employ a variety of different methodologies to assess rail crowding. Some rely on the load factor, while others estimate the rolling hour average loads, or count passengers in excess of capacity [13].

As can be seen in Table 1, the accuracy and precision of the different optical-based solutions listed is claimed to be between 98 and 99 percent in every case. However, these indicators are deceptive, since they are usually obtained under ideal conditions, often in a depot or in a laboratory test. In addition, the accuracy of an estimation is sensitive to camera angle, passenger-flow density, and lighting conditions. If any of these is subject to variation, accuracy will rapidly decline [14].

Researchers have been developing solutions to improve APC’s estimations and passenger count systems based on image and video processing. Nasir et al. [15] developed a methodology for estimating the number of passengers on a bus, which involves processing on-board bus images using several image processing techniques including colour conversion, image segmentation, and the removal of noise. Likewise, some authors [16,17,18] have used image processing for estimating the passenger count by attempting image segmentation on different colour conversions or with the help of depth/thermal cameras. Other passenger counting techniques have been proposed that harness machine learning (ML) and DL algorithms.

**Table 1:** Review of the main European commercial optical APC systems.

Company	Technology	Device	Accuracy—Precision	Source
EUROTECH (Manufacturer)	Stereoscopic vision with IR sensors	DynaPCN 10-01 DynaPCN 10-20-00 DynaPCN 10-20-01	98–99%	[19]
HELLA AGLAIA (Manufacturer)	Stereoscopic vision with IR sensors	APS-R APS-B	98–99%	[10]
IRIS Intelligent Sensing (Manufacturer)	3D IR matrix sensor (ToF)	IRMA Matrix	99%–N/A	[20]
DILAX INTELCOM (Manufacturer)	Triangulation method Stereoscopic vision	DILAX IRS-320R DILAX IRS-400	99%–N/A	[21]
SELSAT (Selecta Digital Service) (System Integrator)	Stereoscopic vision with IR sensors	DynaPCN 10-01	98–99%	[22]
AESYS (System Integrator)	Stereoscopic vision	Aesys VideoSIGHT	98%–N/A	[23]
Retail Sensing (System Integrator)	Optical solution	-	99%–N/A	[24]

Neural networks (NN) have been used in passenger counting systems, notably in the study of Liu et al. [25], where a convolutional neural network (CNN) and a spatio-temporal context model were used to detect passengers and to track their moving heads. Kumar Singh et al. [26] proposed a single shot detector (SSD) mobile net along with a centroid tracker for the improved extraction of features and classifications, while Hsu et al. [27] combined two deep learning methods to estimate passenger occupancy in different scenarios: a convolutional autoencoder to extract features from crowds and determine the number of people, and the ‘You Only Look Once’ version 3 architecture (YOLOv3) for detecting the area of a bus in which head features are clearest. Similarly, Seidel et al. [28] proposed an end-to-end Long Short-Term Memory network for automated passenger counting using a privacy-friendly dataset, only containing depth information from low-resolution 3D LiDAR video recordings; the study claimed an average accuracy of 96% for both boarding and alighting.

A number of applications in the literature combine an object detection algorithm with a tracking algorithm for counting and identifying when a passenger is boarding or alighting. Valencia et al. [29] explored the use of different YOLO algorithms combined with a DeepSORT tracking algorithm for overhead people detection and count; their findings indicated that Tiny-YOLOv4 provides better accuracy with only few false predictions. Meanwhile, Zhang et al. [30] combined two-class SSD model for passenger detection, with a Kalman filter for tracking purposes. They found that DL algorithms yield significantly superior accuracy in comparison to traditional image algorithm methods. Likewise, by combining YOLOv2 and an MIL tracker, Liu et al. [31] proposed an algorithm for counting the number of passengers boarding and alighting in real time, claiming an accuracy of up to 99%. To improve the object detection performance, Zhang et al. [32] proposed a modification on the convolutional layer of a tiny YOLO network to effectively improve the operational speed without compromising the detection accuracy.

Other NN algorithms used in the literature are multi-column CNN for estimating a density map of the passengers’ heads and counting them [33,34]. On the other hand, some authors have combined both ML and DL methods. Nakashima et al. [35] used a YOLOv3 + DeepSORT algorithms to estimate the number of passengers boarding and alighting from a CCTV camera installed on a bus, and then proposed a correction method by applying a Random Forest model. This method improved the estimation by 2.7%: the average accuracy of the image processing estimation was 93.5%, and this rose to 96.2% after application of the correction method. Similarly, other authors have focused on low-cost systems that utilize existing surveillance cameras installed on buses and process videos off-line. These authors have tested different ML and DL algorithms for passenger detection [27,36,37]. In addition, there have been attempts to enhance existing APC estimations: some authors have tested different algorithms for real-time passenger counting [38,39,40,41,42], while others have introduced methods that can be applied in a post-processing phase [43,44].

In the literature, outside the field of transport, there are studies focusing on people detection and counting that employ similar approaches based on overhead video cameras. Some authors have used Kalman filtering for tracking purposes [45] and to improve a YOLO-based object detection and tracking algorithm [46]. Others have used only object detection algorithms for counting [47,48].

Alongside the development of new passenger detection algorithms, there has been some work in developing low-cost APC devices for public transport applications. The cost of a commercial APC is between EUR 1500 and EUR 3000 per door, and generally it is supplied as part of a service with a monthly fee (Information obtained from verbal exchanges with several transport companies). Some devices from the literature have a cost that is half that of commercial products, while providing an equivalent or superior performance in in-field applications. In Sydney (Australia) the metropolitan public bus services conducted an experiment over a period of seven days, including weekdays and weekends, to evaluate different APC solutions. One of these was a real-time-performance video-based passenger counting system [49] with a total cost of approximately AUD 560. This system featured a Raspberry Pi 3B+ as a microprocessor with a Neural Compute Stick connected, and a Raspberry Pi Module V2 camera. Regarding the software, for the detection algorithm, the authors used only MobileNet SSD, a CNN, and the Intersection over Union (IoU) calculated between two consecutive detection bounding boxes as the tracking algorithm. Their system obtained an overall accuracy of 57%. Kniess et al. [50] developed a counting scheme with similar hardware, but they added an aggregation algorithm for managing data-transmission strategies. The detection and tracking algorithm was executed on-board while the classifier ran on an external computer. This system was less successful than YOLOv3 in identifying boarding and alighting by users, although YOLOv3 has a long count processing time (800–1000 s). In contrast, Recalde et al. [51] developed a counting system with a boarding and alighting accuracy of, respectively, 89% and 88%. They performed the data acquisition and the network training entirely on a Jetson Nano, a powerful microcomputer [52].

Although the literature includes the development of both algorithms and low-cost devices, i.e., cost-effective devices that utilize affordable price and computational resources for counting passengers, and although work has been performed on improving commercial APC estimations, there have been only few studies that have used real operational data [16,17,25,27,33,36,37,38,39,40,49,50]. Among these studies using real operational data, none have sought to evaluate commercial APC systems in real-world operational conditions and to compare them with low-cost APC systems.

Considering the significant cost of commercial APC systems, which directly impact the finances of public transport companies, and considering the high levels of accuracy that the manufacturers of these systems claim they provide, companies need to be able to determine how well systems perform in real-world situations, beyond controlled tests. They also need to know how low-cost video-based APC systems perform under similar conditions. A thorough accuracy assessment is essential. Such an assessment should not only examine the performance of commercial APC systems in real operational scenarios, but also highlight differences with respect to a low-cost alternative. This analysis should identify areas of potential improvement for passenger counting in public transport companies. Furthermore, it should help companies to channel their future efforts to optimize the use of resources.

The present paper presents a comprehensive methodology that aims to address two main objectives: (1) assessing the accuracy of a commercial APC system under real operational conditions; and (2) experimentally comparing a camera-based APC system and a low-cost system developed by the authors and installed on a bus.

The paper is structured as follows. The following section presents the methodology to assess the accuracy and the experiment involving the low-cost system, which includes designing the low-cost camera-based APC system, data collection, and analysis. The Results section presents and compares the performances of the two systems. Finally, in the Discussion and Conclusions sections, we seek to position our results in the context of the literature and suggest future research.

## 2. Methodology

To properly assess the accuracy of the commercial APC system and compare it to a low-cost system, a sufficiently large data base of ground truth data from the commercial APC system was needed. To this end, a two-step methodology was designed, involving two in-field tests. The first step was manual acquisition of ground truth data on a bus equipped with the commercial system, provided by Asti Servizi Pubblici (ASP), the public transport company in the city of Asti in the north-west of Italy. One month of ground truth data allowed the accuracy of the commercial system installed on the bus to be checked. The second step was comparing the performance of the commercial system with that of a low-cost camera-based system, and this was performed using a bus run by Gruppo Torinese Trasporti (GTT), the public transport company in the city of Turin, the capital of the region and situated 40 km from Asti. The commercial APC system was the same in the case of both companies, namely ASP and GTT: a camera-based system bought from a commercial supplier.

### 2.1. Assessment of the Accuracy of the Commercial APC System in Asti

The performance accuracy of the commercial APC system was measured against ground truth in regard to three different features: boarding, alighting and vehicle occupancy. The accuracy assessment was performed in the city of Asti, which has about 75,000 inhabitants, with the collaboration of Asti Servizi Pubblici (ASP), the local public transport company that runs a fleet of 64 vehicles, serving 23 urban and suburban bus lines [53].

Data were collected over a four-week period from 19 October 2021 to 19 November 2021, excluding weekends, from 7 a.m. to 4 p.m. Thus, 20 days were monitored, collecting approximately 180 h of data. The bus had 2 doors, and the passengers could board or alight via either of them. Four employees of the start-up Mobyforall (The startup Mobyforall (www.mobyforall.com, accessed on 13 September 2021) kindly provided the data collected by their team for this study) ensured an accurate collection of ground truth data; they were present on board the bus, two for each door. Time stamps, vehicle occupancy, and the number of passengers boarding and alighting at each stop were recorded using pen and paper. Because of certain planning and logistical constraints to which ASP was subjected, the data collection was carried out on a single bus, which was assigned to different lines during the collection period.

ASP then provided the numbers of boarding and alighting passengers as recorded by their APC system, in the form of a database in which each row contained the street name of the stop, along with the total count of passengers recorded as boarding and alighting at that specific stop. Since occupancy information was not included in the provided data, this was calculated during the subsequent data processing phase.

#### 2.1.1. Data Processing

The data from manual counting and from the commercial APC were processed in order to enable a valid comparison:

- Data from manual counting, in the form of a sheet for each door, were digitized and standardized to a single timestamp format;
- Data from the commercial APC only provided information on passengers boarding and alighting at each stop. Any lack of accuracy in this counting causes errors in the calculation of the occupancy between stops. These errors are cumulative, and the overall vehicle occupancy error will increase as a bus continues its journey. To handle this issue, some transport companies have opted to reset the vehicle occupancy at the end of each journey. We calculated vehicle occupancy using two different reset strategies: (i) vehicle occupancy was set to 0 at the beginning of each day; (ii) vehicle occupancy was set to 0 at the start of each journey, taking no account of passengers boarding or alighting at the terminus, because passengers often enter and leave a bus multiple times while it is at the terminus.

#### 2.1.2. Data Analysis and Validation

Different evaluation metrics were used to quantify the performance of the commercial APC system; these metrics express the accuracy of estimations with respect to the ground truth collected during the field study. The aim is to identify the accuracy in terms of boarding, alighting and vehicle occupancy.

To the best of our knowledge [53,54,55], transport companies generally report accuracies in terms of percentages, and, thus, a choice was made to calculate the error by using the symmetric mean absolute percentage error (*SMAPE*), since this has an upper bound of 100% and a lower bound of 0%; in addition, this metric has recently been gaining more popularity, along with the coefficient of determination, within the machine learning community [56]. The error was, therefore, calculated using the *SMAPE* formula presented in Equation (1):

(1) $$SMAPE=\frac{100\%}{n}\sum_{t=1}^{n}\frac{\left|F_{t}-A_{t}\right|}{\left\lceil A_{t}\right\rceil +\left|F_{t}\right|}$$

where n is the total number of observations; $A_{t}$ is the actual value or the ground truth value at observation t; and $F_{t}$ is the forecasted value at observation t.

The accuracy is then calculated using Equation (2):

(2) Accuracy=100%−SMAPE

### 2.2. Low-Cost APC System Design and Test

The test was performed in the metropolitan area of Turin, which has about 1.7 million inhabitants. Gruppo Torinese Trasporti (GTT) is the local public transport company and manages around 929 vehicles serving 91 bus and tram routes [54]. To compare the low-cost passenger counting system with the commercial camera-based APC system used by GTT, a five-step methodology was formulated: (i) design of the low-cost camera-based counting system; (ii) definition of the sampling plan; (iii) 6 days of data collection, for both the low-cost system and the already-installed commercial APC system, in real operational conditions; (iv) data pre-processing and processing for the collected datasets to estimate boarding, alighting and vehicle occupancy; and (v) data analysis and validation.

#### 2.2.1. Design of the Low-Cost APC System

**Figure 1:**
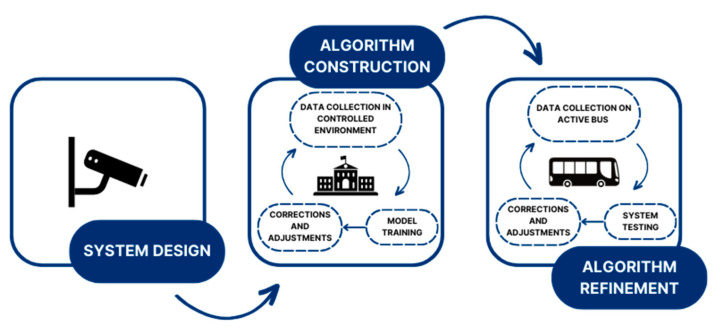
Methodological framework for the low-cost APC system.

The APC system was designed with a primary emphasis on achieving both a low price and good performance. The system developed was composed of the hardware, an object recognition algorithm, and an object tracking algorithm. Figure 1 shows the procedure followed in the design step, including the choice of a camera and a system architecture suitable for our experimental purposes. An initial object detection algorithm was built using data collected in a controlled environment, and this algorithm was subsequently refined using real application data collected in collaboration with GTT on one of their buses.

The main components of the system were the board and the camera. The board was what connected the camera with the video recording and backend system. It also saved the recorded video in its internal memory and, if needed, was capable of running a real-time passenger detection algorithm. The board must, therefore, be powerful enough to handle these operations without any memory or heating issues arising. The camera needs to be compatible with the board system and to be able to produce good-quality images.

- System design

The system was designed in accordance with certain functional and technical criteria: it had to be independent of the bus, not invasive, and have a very low vibration sensitivity. Also, to provide good-quality data, the system needed to have good computational capabilities and enough computational power. The camera needed to be able to record videos at no less than 30 FPS and with a good resolution [49].

The Raspberry Pi 3 Model B was a suitable “universal” board, allowing any of the three cameras considered to be connected without any technical issues. It comprises a quad-core 1.2 GHz Broadcom BCM2837 64-bit CPU, 1 GB RAM, a CSI camera port, and a Micro SD port for extra memory space. Its operating system is Raspberry Pi OS (previously called Raspbian), a Linux-based OS including basic programs, utilities, numerous packages, and pre-compiled software [57]. Raspberry Pi is, thus, a mini-PC with a computational capacity enabling it to execute Python scripts and lightweight object recognition models [5,58,59,60], making it a good choice as the system motherboard.

**Figure 2:**
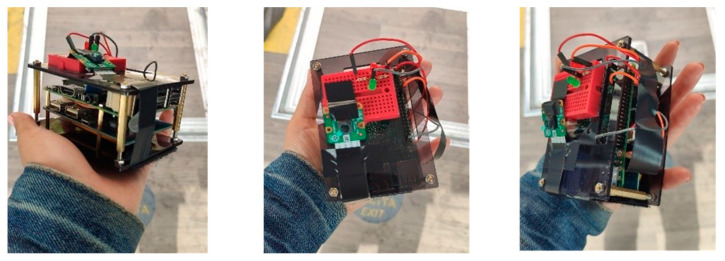
Low-cost APC system.

Three different cameras were considered: an SPI camera, a Raspberry Pi camera, and a HIMAX CMOS HM0190. The Raspberry Pi camera offered the best resolution, with the possibility of changing and regulating the resolution if needed. The image resolution, frame rate and board worked well together as a system, providing a good level of accuracy [61,62,63], and this is why the Raspberry Pi camera was chosen. Figure 2 shows the low-cost APC system assembled.

- Algorithm construction and refinement

Since this experiment was performed in a real setting, it was important to set up and test the system in a controlled environment before going in-field, in order to know the optimal conditions for its deployment. The most significant factors are (1) the height at which the camera is placed, as this can cause detection issues; (2) the camera angle, essential for ensuring passenger privacy; and (3) the lighting conditions. The controlled experiment plays a crucial role in highlighting any weak points, and it is essential that the simulated conditions reflect as far as possible the real in-field conditions. To this end, the controlled experiment included the following:

1. The simulation of different lighting conditions;
2. The simulation of different levels of crowdedness;
3. The simulation of people’s different boarding and alighting patterns;
4. The simulation of people wearing and carrying accessories.

The controlled experiment was performed using a door at the entrance to a study room inside the main campus of the Politecnico di Torino. The door provides access from the exterior, so it was possible to obtain different lighting conditions, and moreover it is a door used by a large number of students wearing or carrying a variety of accessories. Entering the study room was considered to be ‘boarding’, and exiting was considered to be ‘alighting’. In total, 10 h of data were collected, 70% of which was used as input for training the algorithm, and the remaining 30% for testing it. To this end, a pre-processing step was necessary to extract the frames to be used for the input dataset. The training set of the frames was then labelled, and the weights were trained.

The following step concerned the object detection algorithm. YOLO is a one-step detector which has the advantages of high accuracy, good learning capabilities and a speed that allows real-time applications; it is three orders of magnitude faster than R-CNN while exhibiting good object detection [64], and the literature indicates that one-step detectors represent a good compromise between object detection performance and computation time [65]. After having trained different versions of YOLO, we chose YOLOv5m [66,67,68], which provided the best results, with Precision = 0.95, Recall = 0.89, mAP@.5 = 0.96, mAP@[.5:.95] = 0.62. Next came a step relating to the tracking and counting stage, for which the DeepSORT algorithm was considered the best option, as it is a multi-object tracking algorithm widely used in the transport sector and has been shown to have good accuracy [69,70,71].

The tracking and counting algorithm does not require a training phase, since it uses the pre-trained weights of the previous step as the input. The extent to which the tracking phase is successful is highly dependent on the confidence level achieved in the detection of each subject, avoiding ID losses and re-associations with a person already detected in the previous frame. The tracking phase leads on to the actual counting implementation, which consists of drawing two regions of interest (ROI) over the frame: the width and location of the ROI are determined according to the area of the frame where the tracking results are the most effective.

For our low-cost APC system to work as intended in a real setting, a refinement step was necessary; in-field data were collected to re-train the algorithm.

#### 2.2.2. Sampling Plan and Experimental Set up

Different requirements had to be met to ensure the dataset heterogeneity necessary for preventing irregular behaviours of the algorithms, and avoiding overfitting and underfitting. The collected dataset needed to be statistically representative of the population under study. We, therefore, paid particular attention to how training data for the in-field scenario were acquired, and used a sampling plan to ensure its representativeness. The formula shown in Equation (3) was used to calculate the required sample size, according to the literature [72]:

(3) $$n=\frac{{\left(z\right)}^{2}\times p(1-p)}{{∆}^{2}}$$

where n is the sample size; z is the level of confidence according to the standard normal distribution (for a level of confidence of 95%, z = 1.96, for a level of confidence of 99%, z = 2.575); p is the estimated proportion of the population that presents the selected characteristic; and Δ is the accepted error (accepted error percentage, not exceeding 5%). In our case, the sampling unit is the passenger and p is set to 0.5.

Given the accepted error and the corresponding sample size, it was a question of determining how many days of recordings were needed to achieve the desired results. To this end, the formulas shown in Equations (4) and (5) were used:

(4) $$n=p_{ride}\times {ride}_{day}\times days$$

(5) $$days=\;\frac{n}{p_{ride}\times {ride}_{day}}$$

where n is the sample size; $p_{ride}$ is the average number of people recorded during one journey; ${ride}_{day}$ is the number of journeys considered in one day of recordings; and days (the target variable to be determined) is the number of days needed for data collection.

The level of error selected was 2%, requiring us to count more than 2400 passengers. Since an average of 60 people ($p_{ride}$ = 60) are transported per journey, and considering 4 journeys per day by applying the formula of Equation (5), we collected data over 10 days in order to achieve representativeness. As each journey takes around one hour, the total number of recording hours obtained was 40 h, corresponding to more than 5000 videoclips of data. The frames from these clips were labelled and used for re-training the weights in the algorithms.

The test was carried out aboard a bus belonging to GTT. Due to constraints related to planning and the availability of GTT personnel, the trial was performed in a single bus and involved manually counting passengers boarding and alighting over three days in 2022: 13, 30, and 31 May. Following the analysis of preliminary results, it was decided that three additional days of data would be required for a better understanding, and this was collected on 3, 7, and 9 November. We were, thus, able to compare passenger numbers obtained by the commercial APC, by our low-cost system, and by our manual count (the ground truth).

**Figure 3:**
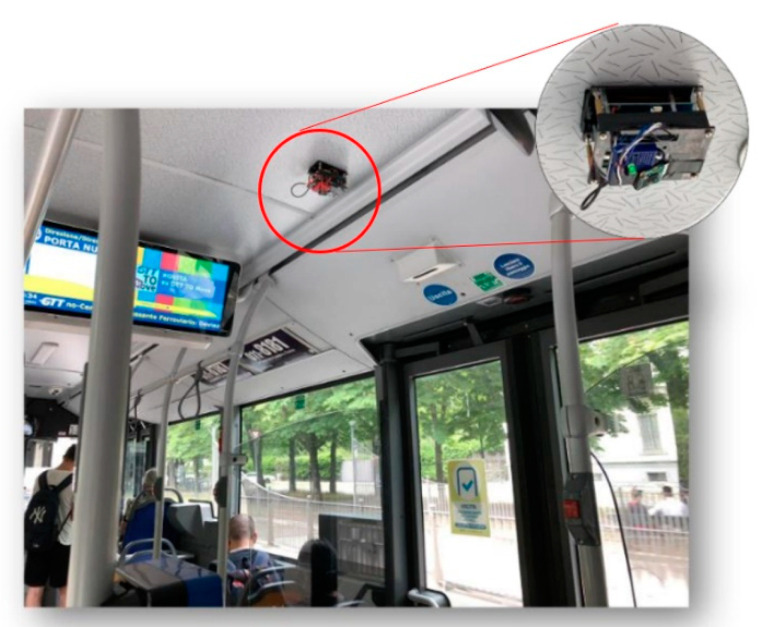
Low-cost APC installation.

**Figure 4:**
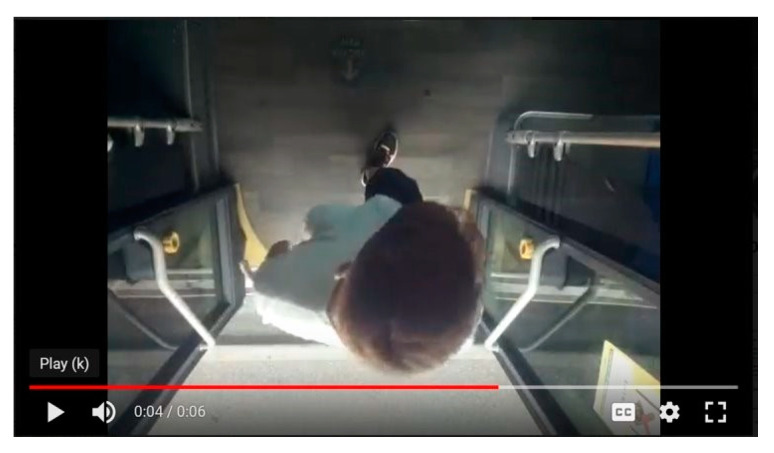
Low-cost APC camera video frame.

The bus assigned to the experiment has three doors that passengers can use for both boarding and alighting. The commercial APC system already installed on the bus by the transport company is a stereoscopic camera that counts the number of passengers boarding and alighting at each bus stop. Our low-cost device is small enough to be classified as non-invasive. A camera was located above each door so as not to point directly into passengers’ faces (Figure 3). The passenger privacy stipulated by the company was, thus, ensured, since facial images were not recorded (Figure 4). The camera was activated manually to record a video every time the door was opened.

#### 2.2.3. Data Processing, Analysis and Validation

**Figure 5:**
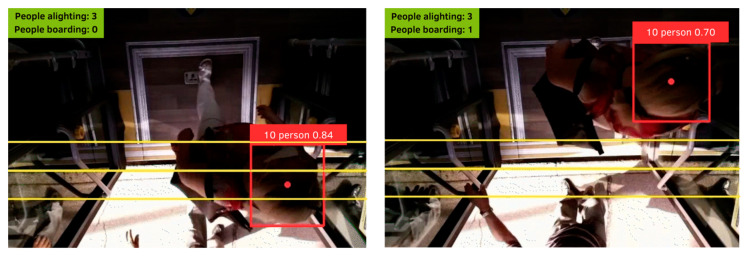
Output of the algorithm in the low-cost APC.

Each of the three systems had its own procedure:

- For the manual count and the commercial APC, the processing was very similar to the one described in Section 2.1.1. Vehicle occupancy had already been estimated from the data provided by GTT, and some cleaning and imputation were done, since there were empty fields relating to vehicle occupancy, and some stop-related information needed to be filled in.
- The low-cost system required the use of the algorithm to estimate the different values. The frames of the videos at each stop were used to initially estimate the number of passengers boarding and alighting. The vehicle occupancy was then calculated for the testing days. Figure 5 shows the output of the algorithm; the person is identified and assigned to a centroid that is tracked over the video. The ROI lines indicated the direction of the passenger, that is to say, whether they were boarding or alighting.

The evaluation metrics applied are the same as those presented in Section 2.1.2.

## 3. Results

This section first presents the results of the accuracy assessment. Secondly, the performances obtained on the experiment for both the commercial system and the low-cost device are compared.

### 3.1. Assessment of Accuracy of Commercial APC

The accuracy of the commercial APC system was found to be 53.17% for boarding and 55.29% for alighting. When resetting the calculated vehicle load once per day, the accuracy of the commercial APC was found to be 21.24%. Yet, the accuracy when resetting the vehicle load to zero at the beginning of each new journey increased to 57.74%.

**Figure 6:**
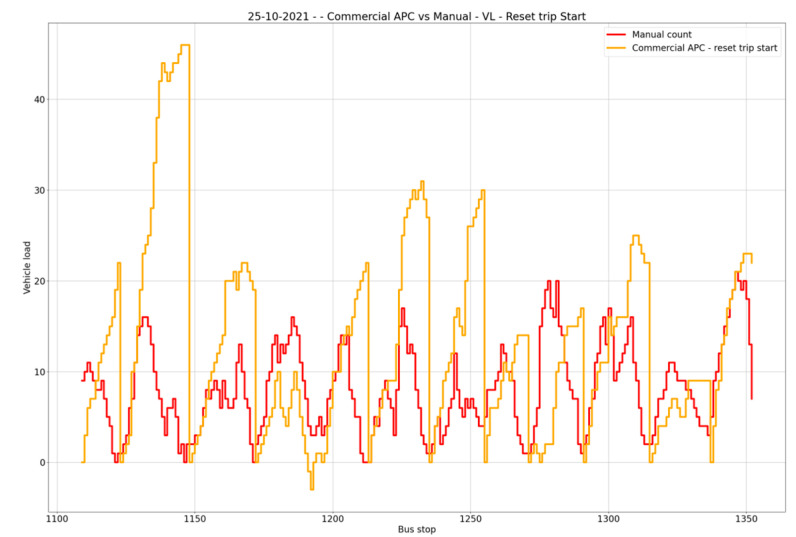
Highest overall accuracy: 25 October 2021.

**Figure 7:**
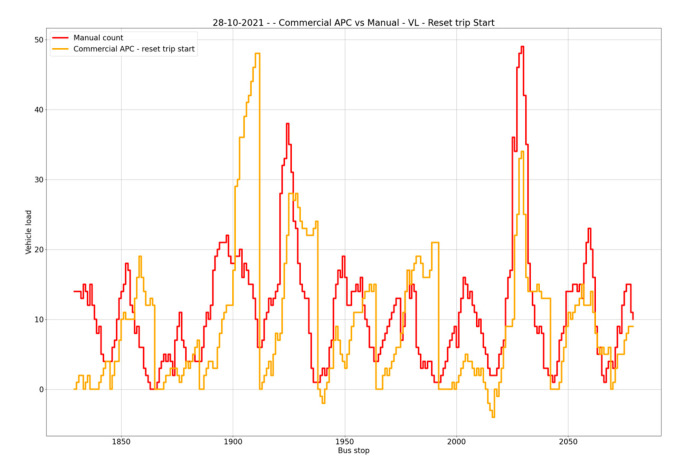
Lowest overall accuracy: 28 October 2021.

When looking at accuracy for individual days, as shown in Figure 6, the highest overall accuracy was achieved for 25 October 2021, with a vehicle occupancy accuracy of 60.40%; on that day, the accuracies for boarding and alighting were, respectively, 56.25% and 54.67%. The worst accuracy obtained was for 28 October 2021, with 53.78% for vehicle occupancy, 50.32% for boarding and 51.33% for alighting, as shown in Figure 7.

In some cases, the calculated vehicle occupancy fell below zero due to errors in the numbers of passengers boarding and alighting, as counted by the APC. Overall, the percentage accuracy of the commercial system was between 50% to 65% when considering individual journeys.

### 3.2. Results of the Test on Two APC Systems

Although the data collection was carried out over six days, GTT provided data files only for two days, 30 and 31 May 2022, since there had been a malfunction in the commercial APC on the other days. We, therefore, decided to test both systems only for the days for which GTT provided the data. Over the two days, the bus was assigned to two different routes. On day 1 it was operating on the periphery of the metropolitan area (on what we have called the “uncrowded line”), while on day 2 it was operating on a route (the “crowded line”) running through the tourist district and past schools and an outdoor market.

During the analysis, we realized that some data from the commercial APC were lacking for the period in which the manual count was being conducted. Thus, a direct comparison between the commercial and low-cost systems was performed only for day 1, while the low-cost APC was evaluated in relation to the manual count over the whole six days. The accuracy results for the two systems are reported in Table 2.

#### 3.2.1. Low-Cost APC

**Figure 8:**
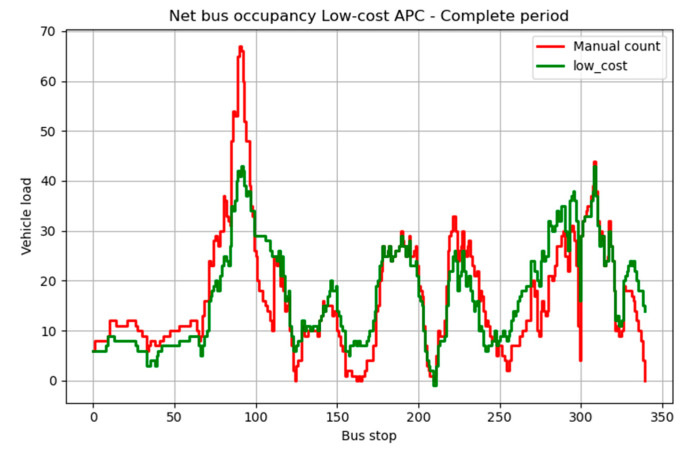
Vehicle occupancy for the low-cost APC system over the whole 6-day period.

The overall performance of the low-cost system was much better than expected. The low-cost system achieved an overall accuracy of 81.59% for vehicle occupancy, 72.27% for boarding passengers, and 74.59% for alighting passengers, as reported in Table 2. Furthermore, the distribution of vehicle occupancy was very similar to that found by the manual count (Figure 8).

The performance was better on the uncrowded line, particularly regarding boarding and alighting passengers, with an accuracy, respectively, of 83.53% and 94.87%. On the crowded line during the peak hours, the algorithm had difficulty counting the number of passengers when they were arriving in large numbers. The highest peak observed in Figure 8 corresponds to when the outdoor market was active and when pupils were returning home after school.

#### 3.2.2. Commercial APC

Due to the lack of data for one of the days, resulting from a malfunction in the commercial APC, we were unable to carry out an evaluation on the crowded line for day 2. On the uncrowded line, the commercial device failed to exhibit the accuracy specified in the product datasheet. As reported in Table 2, the accuracy for boarding and alighting passengers was, respectively, 77.69% and 83.33%.

**Figure 9:**
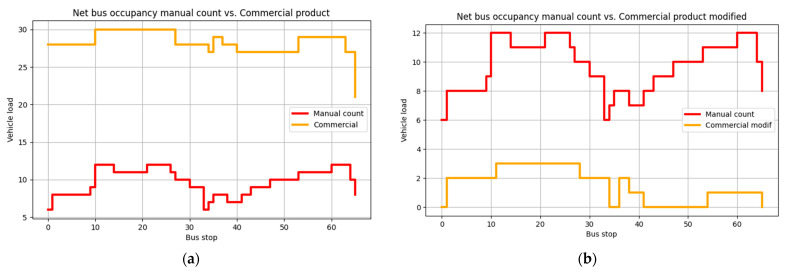
Vehicle occupancy (**a**) with original data and (**b**) with recalculated value.

The accuracy of vehicle occupancy was the lowest, with a value of 50.94%. The poor performance in relation to vehicle occupancy was the consequence of an accumulation of erroneous estimations from previous journeys. For this reason, there was a recalculation of the vehicle occupancy in which the value was reset to 0 when a new journey started. Despite this recalculation, the new vehicle occupancy accuracy was 26.92%. Figure 9 shows the bus occupancy with and without recalculation, and we remark that the original value (before recalculation) is closer to the ground truth pattern distribution.

#### 3.2.3. Comparison between the Systems

**Figure 10:**
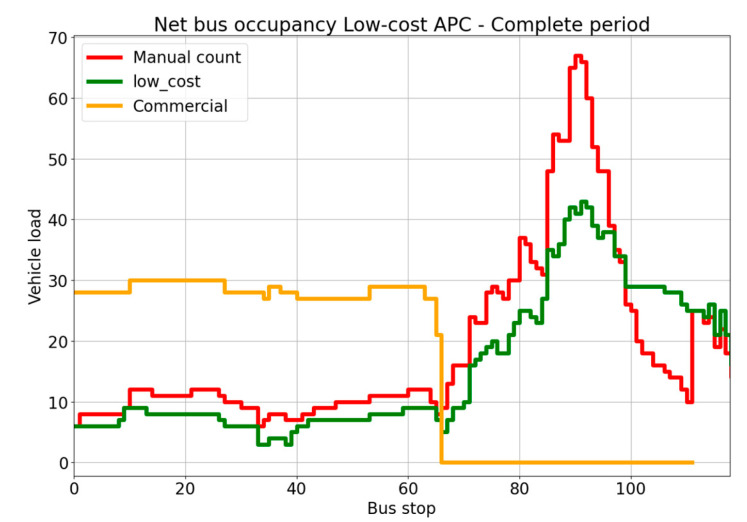
Vehicle occupancy for the three counting systems for the 2-day period.

**Figure 11:**
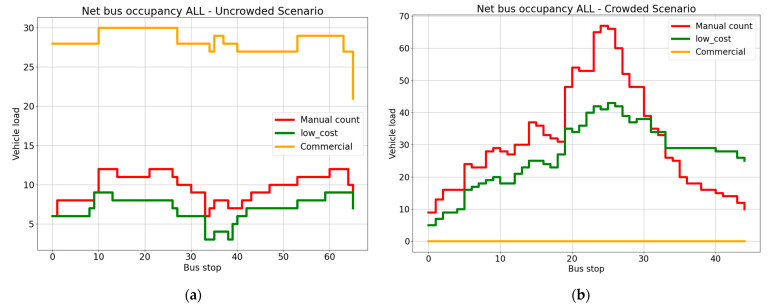
Vehicle occupancy for the three counting systems on (**a**) the uncrowded line and (**b**) the crowded line.

The comparative performance of the two systems was evaluated in relation only to the uncrowded line. As shown in Table 2, the overall performance of the low-cost device was better than that of the commercial APC. The low-cost device obtained a considerably higher accuracy for vehicle occupancy (a difference of 31.52 percentage points with the commercial APC), while the percentage point differences for boarding and alighting were 5.84 and 11.54. This trend is also evident in Figure 10, where the vehicle occupancy estimation by the low-cost system closely aligned with the ground truth throughout the evaluation period, while the estimation by the commercial APC was nearly double the value of the manual count, as shown in Figure 11a.

From Figure 11 we see that the low-cost system has a closer correspondence to the ground truth in both scenarios, confirmed by the correlation values. The highest correlation (0.88) is for the vehicle occupancy obtained by the low-cost system. The lowest correlation (0.062) is for the alighting passengers counted by the commercial APC. Where there is a strong correlation with the manual count, the collected data provide an opportunity for improving the accuracy of the estimations via a correction model. The values estimated by the low-cost system are such that a correction model may potentially be used to advantage in making them more reliable.

## 4. Discussion and Conclusions

Different image- and video-based algorithms for passenger flow counting have been documented in the literature, including some implemented on low-cost APC devices, but no previous study has included an assessment of the accuracy of a commercial video-based APC system. To the best of our knowledge, there have been no performance comparisons with low-cost video-based APC products. In this study, we evaluated a commercial APC system and developed a low-cost video-based APC system, using YOLOv5 + DeepSORT as the detection and tracking algorithm, and with the collaboration of the local public transport company we carried out an in-field experiment to compare its performance against that of a commercial APC system. The study was conducted in two steps: (1) ground truth and APC data were collected over a period of 20 days in Asti to assess accuracy, and (2) a low-cost APC system was developed and implemented, followed by 6 days of data collection on a bus operating in Turin. The performance evaluation made in Asti shows that the estimation of the accuracy of vehicle occupancy of the commercial APC system, after some post-processing, reached a value of 57.74%. For boarding and alighting, the accuracy was found to be below 55%. The low-cost APC system, however, during the 6 days of data collection in Turin, achieved markedly better results. The accuracy of vehicle occupancy was 81.59%, while the average accuracy for boarding and alighting was 74%. The results show that the overall accuracy of the low-cost system was higher by approximately 20 percentage points compared to the commercial APC system assessed in Asti.

The data collected over 6 days in Turin were limited due to malfunctions in the commercial APC system, resulting in only one day of data from the uncrowded line being provided by the transport company. With the available data, the results show that the low-cost device had better accuracy overall than the commercial APC already installed on the bus. The vehicle occupancy estimated by the low-cost system had an accuracy of 82.46%, while that estimated by the commercial APC had an accuracy of 50.9%. For boarding and alighting, the low-cost device had accuracies, respectively, of 83.5% and 94.8%, while the commercial APC had accuracies of 77.6% and 83.33%. Although a direct comparison between the two APC systems in Turin under crowded conditions was not feasible, valuable insights can be extracted from the data gathered in Asti concerning the performance of the commercial APC system on crowded lines. As the number of passengers of public transport in Turin is higher, being a bigger city than Asti, we can presume that the accuracy in Turin could potentially be lower.

Regarding the performance, the low-cost system had the lowest accuracy during peak hours, more obviously in the case of a crowded line, while the commercial system had a very low accuracy in relation to occupancy since it accumulated boarding and alighting errors along the route. Our findings complement those from the literature, confirming that there is a higher error level for net bus occupancy during peak hours [8,27,37,49].

The results indicate that there is a potential for using low-cost APC systems, which represent a good compromise between cost and performance However, there are technicalities to be addressed before this system may be officially adopted. Certified compliance with a number of licenses and standards is required for any device implemented in a public transport vehicle, and consideration is currently being given to a possible future integration of the low-cost system with the transport company’s existing GTFS. Ultimately, all this would have a cost that has not, so far, been included in the cost of our device.

Additionally, the implementation of a secure protocol for transmitting videos or frames over the network is necessary; the computational power of the RaspberryPi 3B is not enough to run an object detection and tracking algorithm smoothly without encountering functional issues. Also, the collected dataset could be utilized to improve the estimation of the accuracy of APC during a post-processing phase [49]. Nevertheless, our experiment highlights the potential of deep learning models in improving the accuracy of both real-time and post-processing schemes. This is, to our knowledge, the first study to demonstrate the potential of a low-cost APC system by comparing it with a traditional APC system in a real-world setting.

This experiment also raises concerns about the performance of current commercial APC systems. Although the formula used to evaluate accuracy was not the formula specified in VDV recommendation 457-2, there was a discrepancy between the measured accuracy (57.74%) of the commercial APC and the accuracy claimed on the product’s datasheet (see Table 1). By comparing the two APC systems, it is evident that the estimation of the commercial APC system is susceptible to many variabilities; from the development of the low-cost system and the subsequent collection of ground-truth data, we could understand and postulate that the video-based APC systems can be affected by the following external factors: (i) the boarding or alighting of large group of passengers may cause over- or under-counting [16,28,40,49]; (ii) passengers who step off (alighted) the bus and immediately re-board could lead to duplicated counts [37]; (iii) when the bus reaches the full capacity, the system faces difficulty in accurately counting passengers due to the crowd around the door [36]; (iv) passengers lingering at the door during the entire trip can lead to inaccuracies [28]; (v) technical settings such as positioning [37,49], resolution quality [25], lens cleanliness and device calibration of the device can impact accuracy; and (vi) the possibility for the commercial APC system to miss some passengers because it is switched off or re-started at each terminus when the bus stays idle. Factors like lighting conditions have affected the results in some studies [25,27,28,37], though the results of our model were not significantly affected by different lighting conditions. Similarly, the presence of large elements [28] such as bicycles or baby carriage, or wearable objects [50], i.e., hats or backpacks, did not significantly affect the estimation of our low-cost APC system. This could be attributed to the use of multiple frames in our training data that included different lighting conditions and types of elements.

Despite our limited knowledge of the manufacturers’ image processing algorithms (not disclosed), our hypothesis suggests that sub-optimal performance may be related to insufficient updates to their estimation software and a lack of proper maintenance. An additional plausible factor, as observed during our ground-truth data collection, could be attributed to the positioning of the commercial APC system in proximity to the bus door. This particular area of the bus exhibited greater vibrations on poor road surfaces compared to the ceiling-mounted location of the low-cost system. Hence, this study shows that it would probably be worthwhile to invest in improving the accuracy of the commercial APC systems, be it through improvements to image processing algorithms or evaluation about placement of the cameras inside the bus. The parameters of occupancy, boarding and alighting are very important because they are key indicators of the performance of the transport networks. By ensuring that these values are in line with actual scenarios, the quality of service can be significantly improved. Therefore, we believe that transport companies and APC system manufacturers should invest in improving the performance of existing systems.

Future work should, therefore, be devoted to exploring the significant potential for increasing the estimation of APC accuracy. Given the impressive rate at which object recognition and tracking algorithms are being improved, there is considerable potential for increasing estimation accuracy by applying and testing algorithms developed in the literature in actual in-field experiments. Investigations into the placement of the cameras within the bus and their susceptibility to vibrations might also be conducted, exploring their impact on the accuracy of passenger counting. This analysis could aid in determining the most suitable camera placement.

In addition, new hybrid methodologies might be explored with a view to developing more robust low-cost real-time passenger counting systems. One such approach could involve the implementation of edge-cloud object recognition models, where a portion of the estimation occurs onboard while another part takes place in the cloud [7]. An alternative strategy involves the testing and adoption of various security protocols for transmitting frames or videos. This aims to leverage superior external computational power (cloud) and consequently attain more precise estimations [73].

Future work could also focus on evaluating other types of commercial APC systems and testing them against a low-cost system in real-world conditions under different crowding scenarios for longer periods.

## Figures and Tables

**Table 2:** Accuracy results from both systems.

APC System	Boarding	Alighting	Vehicle Occupancy
Low-cost APC system			
Overall accuracy in six-day period	72.27%	74.59%	81.59%
Uncrowded line	83.53%	94.87%	82.46%
Crowded line	65.83%	66.68%	80.38%
Commercial APC system			
Overall accuracy Asti’s twenty-day period	53.17%	55.29%	57.74%
Uncrowded line	77.69%	83.33%	50.94%
Crowded line	-	-	

## Data Availability

The datasets generated during and analysed during the current study are not publicly available due to sensitive business information belonging to ASP and GTT that is not intended for public dissemination.
